# Prevalence of HPV infection and other risk factors in a Fijian population

**DOI:** 10.1186/1750-9378-9-14

**Published:** 2014-04-28

**Authors:** Sunia Foliaki, Naomi Brewer, Neil Pearce, Peter JF Snijders, Chris JLM Meijer, Lepani Waqatakirewa, Gary M Clifford, Silvia Franceschi

**Affiliations:** 1 Centre for Public Health Research, Massey University, PO Box 756, Wellington 6140, New Zealand; 2 London School of Hygiene & Tropical Medicine, London, UK; 3 Vrije Universiteit Medical Center, De Boelelaan 1117, 1081 HV Amsterdam, The Netherlands; 4 Ministry of Health, Yaren, Nauru Island; 5 International Agency for Research on Cancer, Lyon, France

**Keywords:** Human papillomavirus (HPV), HPV genotypes, Cervical neoplasia, Cervical screening, Fiji

## Abstract

**Background:**

Cancer is among the leading contributors to morbidity and mortality in the Pacific, but the magnitude of the problem and the potential for prevention have not been comprehensively studied. Over the past decade, cervical cancer has been the most common cancer among women in Fiji with an age standardised cervical cancer incidence rate of 51 per 100,000. This rate is among the highest in the South Pacific region and in the world. This high cervical cancer incidence rate is likely linked to the low cervical screening rate, but it points also to the possibility of a high burden of human papillomavirus (HPV) infection.

**Methods:**

We conducted a population-based survey in Fiji to provide information on human papillomavirus (HPV) prevalence, and the distribution of individual HPV types in a Fijian health-sub-district. We included 1,261 women aged between 16 and 64 years. A general primer GP5+/6+ mediatedpolymerase chain reaction (PCR) assay was used for HPV testing of 44 HPV types.

**Results:**

The crude HPV prevalence in 1,244 women with an adequate HPV sample was 24.0% (95% confidence interval (CI), 21.7-26.4%) and the corresponding age standardised prevalence was 25.5% (95% CI, 23.1-28.1%). The prevalence of high-risk HPV types was 13.6% (95% CI, 11.8-15.6%). Among 1,192 women with adequate cytological results, 13 (1.1%) showed cervical abnormalities, the majority of which were high-grade intraepithelial lesions or worse. HPV prevalence declined from 35.8% in women aged <25 years to 18.6% in those aged 55–64 years of age. After adjustment, the only variables significantly associated with HPV-positivity were age (ranging from odds ratio (OR) 0.57 (95% CI, 0.36-0.89) for 25–34 year-old-women to OR 0.43 (95% CI, 0.20-0.89) for 55–64 year-old-women) and ‘husband’s extramarital sexual relationships’ (OR 1.69; 95% CI, 1.17-2.34).

**Conclusion:**

These findings on HPV provide key information for future policy decisions on the most appropriate methods of cervical cancer prevention in Fiji and in the Pacific region.

## Background

There has been considerable interest and research on non-communicable diseases throughout the South Pacific over the last 40 years [1]. This has not been the case with cancer research however, and there have been relatively few publications [2]. Cancer is among the leading contributors to morbidity and mortality in the Pacific [3], but the magnitude of the problem, the key risk factors, and the potential for prevention have not been comprehensively studied [4]. The patterns of cancer vary widely with some Pacific islands having high incidence rates for infection-related cancers, such as cervical and liver cancers, while others demonstrate a more westernised pattern of cancer prevalence, predominantly colon and breast cancers [4].

The Fiji Islands consist of 322 islands and 840,201 inhabitants [5]. The British colonised Fiji in 1874 and the Indian labourers brought for the sugar cane plantations now account for 43% of the population, with indigenous Fijians (partly Tongan and Melanesian ancestry) accounting for just over 50% [5]. Life expectancy is similar for both Indian and indigenous Fijians (64 years in males; 68 years in females) [5].

Over the past decade, cervical cancer has been the most common cancer among women in Fiji with an age standardised cervical cancer incidence rate of 51 per 100,000 [4]. This rate is among the highest in the South Pacific region [6] and in the world [7]. This high cervical cancer incidence rate is likely linked to the low cervical screening rate, but it points also to the possibility of a high burden of human papillomavirus (HPV) infection.

HPV genotyping of cervical cancer and cervical intraepithelial neoplasia specimens taken in 2003 to 2007 from 296 women at Fiji’s Colonial War Memorial Hospital, showed that HPV16 and 18 were the most common genotypes (77%), followed by HPV 31 (9%) and HPV 52 (7%) [8]. However, there have been no studies of the general population prevalence of HPV in Fiji, and only one elsewhere in the Pacific – in Vanuatu in 2009-2010 [9].

We therefore conducted a study to determine the prevalence of HPV infection, cervical abnormalities, and other cervical cancer risk factors among a sample of women in the Valelevu health-sub-district of Suva, the Fijian capital. The study was a collaboration between Massey University and the International Agency for Research on Cancer (IARC), in partnership with the Fiji Ministry of Health’s Cervical Cancer Screening Programme.

## Results

A total of 1,261 women aged 16–63 years (mean 36.9 years) participated in the study, and 944 (75%) lived in the study area. All 1,261 women provided a sample for HPV analysis, 17 were beta-globin negative, leaving 1,244 available for HPV analysis. A total of 1,192 cervical samples were adequate for, and assessed for, cytology.

Table 1 gives the findings for the prevalence of the various HPV types in 1,244 women for whom a valid HPV result was obtained. The overall crude prevalence of HPV infection was 24.0% (21.7-26.4%) and the corresponding age standardized prevalence was 25.5% (95% CI, 23.1-28.1%). The corresponding estimate for HR types was 13.6% (11.8-15.6%), and that for LR types was 14.9% (13.0-17.0%). HPV 16 was the most common type (4.0%; Table 1). Among HR types, HPV 16, 52, 56 and 59 were each found in ≥1.5% of women overall. HPV 18 was found in 1.1% of women. Among LR types HPV 42 was the most common (3.0% overall), while 66, 70, 81 and Jc9710 were each found among ≥1.4% of women. HPV 6 and 11 were detected in 0.5% and 0.2% of women, respectively. Multiple HPV types accounted for 29.9% of HPV-positive women.

**Table 1:** Prevalence of human papillomavirus (HPV) types among 1,244 women, Fiji 2010-2011

**HPV type**	**Total (n = 1,244)**^**a**^			
	**Single**	**Multiple**	**Total**	**(%)**
HPV-	-	-	946	76.0
HPV+	209	89	298	24.0
High-risk HPV+	141	28	169	13.6
Low-risk HPV+	151	34	185	14.9
High-risk infections				
16	43	7	50	4.0
18	9	5	14	1.1
31	9	6	15	1.2
33	1	4	5	0.4
34	0	0	0	0.0
35	10	3	13	1.0
39	4	2	6	0.5
45	9	8	17	1.4
51	7	3	10	0.8
52	15	4	19	1.5
56	17	10	27	2.2
58	6	0	6	0.5
59	10	12	22	1.8
68	1	2	3	0.2
Low-risk infections				
6	4	2	6	0.5
11	1	2	3	0.2
26	0	1	1	0.1
30	2	3	5	0.4
32	1	2	3	0.2
40	2	4	6	0.5
42	28	9	37	3.0
43	6	7	13	1.0
44	1	0	1	0.1
53	5	4	9	0.7
54	2	2	4	0.3
55	1	1	2	0.2
61	1	0	1	0.1
64	0	0	0	0
66	15	3	18	1.4
67	10	2	12	1.0
69	1	0	1	0.1
70	16	4	20	1.6
72	3	0	3	0.2
73	0	1	1	0.1
81	9	8	17	1.4
82	6	3	9	0.7
83	5	1	6	0.5
84	3	1	4	0.3
85	3	1	4	0.3
86	3	1	4	0.3
97	0	0	0	0.0
Cp6108	5	1	6	0.5
Jc9710	14	12	26	2.1
Low-risk unspecified	4	0	4	0.3

^a^68 inadequate cytology are included in the total.

**Figure 1:**
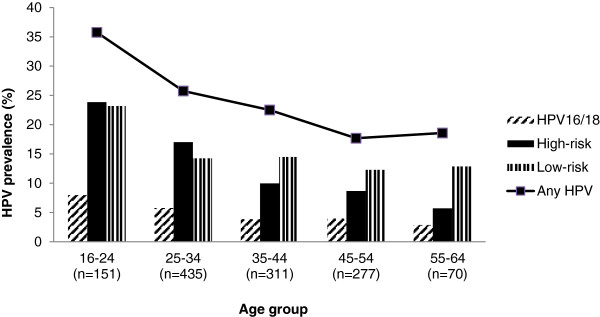
Age-specific prevalence of human papillomavirus (HPV) by HPV types.

Figure 1 shows the age-specific prevalence of HPV in 1,244 women (i.e. all of the women for whom a valid HPV result was obtained), 298 of whom were HPV-positive, classified into: (i) HPV 16/18; (ii) any HR; (iii) any LR; and (iv) any HPV genotype. Prevalence decreased from 35.8% in women aged less than 25 years to 18.6% in women aged 55–64 years. Similar patterns were observed for each group of HPV types. HPV16 and 18 accounted for 36.7% of HR HPV-positivity in the general female population, ranging from 33.3% among women aged 16-24 years to 50.0% among women aged 55–64 years.

Among the 1,244 women who had a valid HPV result, 39 (3%) had inadequate Pap smear results. Among women with adequate cytological results, cervical abnormalities were diagnosed in 13 women, of whom 84.6% were HPV-positive. The proportion of cytological abnormalities varied from 1.7% in women aged 25–34 years, down to 0.34% in women aged 35–44 years.

Cytological abnormalities included four ASCUS (one HPV-negative; 16 and 18; 81, Jc9710 and 52; 66), one low-grade abnormality (HPV45), one atypical squamous cells - cannot exclude high-grade squamous intraepithelial lesion (ASC-H) (HPV 42), and six high-grade abnormalities, (one HPV-negative; HPV 6 and 16; 6 and 52; 53 and 51; 66 and 58; 56), and one clinically detectable invasive cervical cancer (HPV16). All of the women with cytological abnormalities were recalled for follow-up.

**Figure 2:**
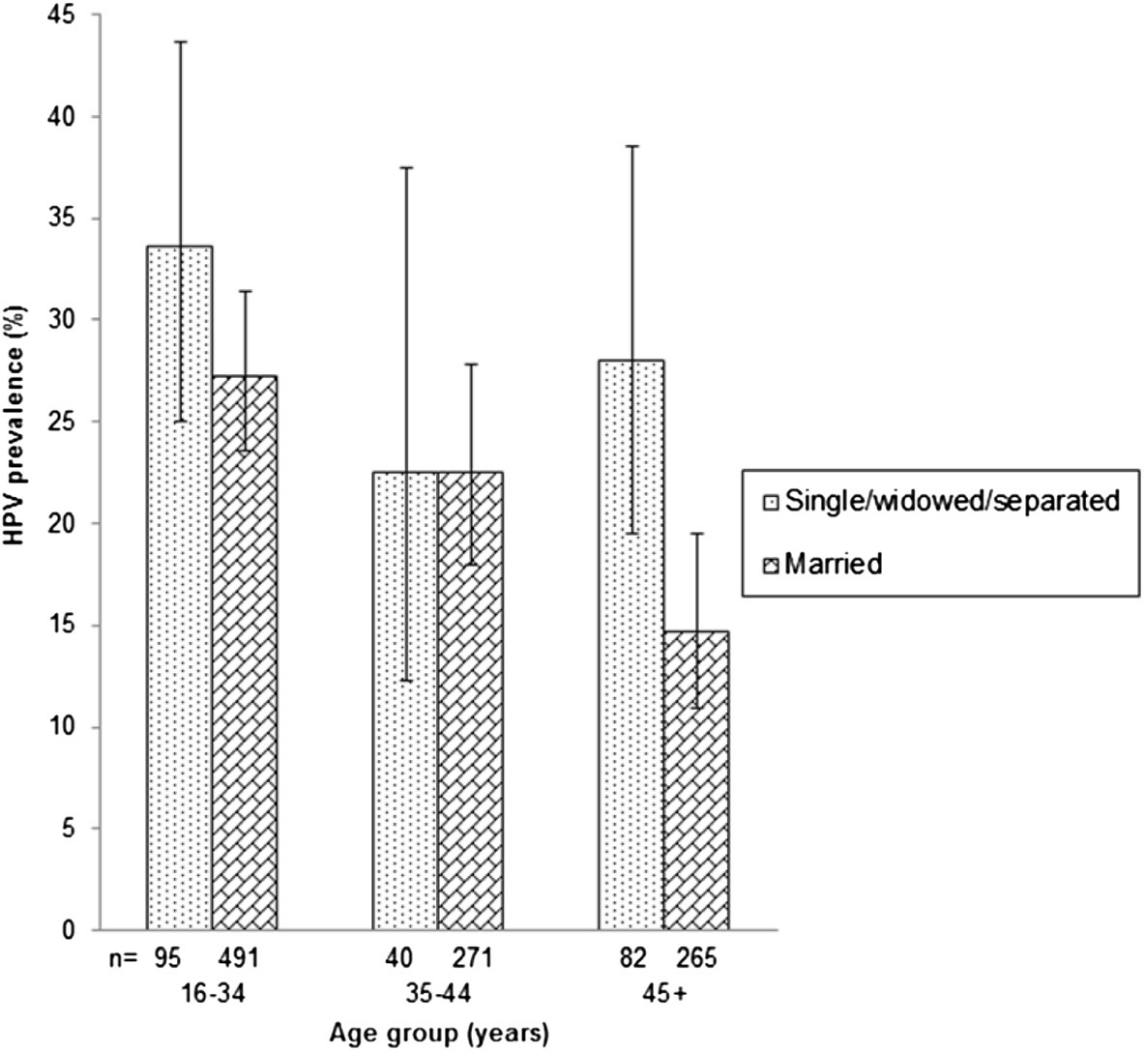
Age-specific prevalence of human papillomavirus (HPV) positivity by marital status.

Table 2 shows the relationships between HPV positivity and various potential risk factors. The only statistically significant positive associations were for separated/divorced/widowed *versus* currently married women (OR = 1.68; 1.04-2.73) and report of husband’s extramarital sexual relationships (OR = 1.69; 1.20-2.38). A non-significant positive association was found between HPV positivity and nulliparity (compared with one full-term pregnancy) but no significant difference by number of full-term pregnancies. The association with being separated/divorced/widowed was mainly driven by findings in women aged 45 or older (Figure 2). It was greatly diminished by adjustment for husband’s extramarital sexual relationships (Table 2).

**Table 2:** Odds ratios (ORs) for human papillomavirus (HPV) positivity and corresponding 95% confidence intervals (CIs) according to selected risk factors

**Risk factors**	**No. women**	**HPV-positive women**	**(%)**	**Age-adjusted OR**	**95% CI**	Multivariate OR*	**95% CI**
**Age-group**							
16–24	151	54	35.8	1	–	1	–
25–34	435	112	25.8	0.62	0.42–0.93	0.57	0.36–0.89
35–44	311	70	22.5	0.52	0.34–0.80	0.50	0.31–0.81
45–54	277	49	17.7	0.39	0.25–0.61	0.37	0.22–0.61
55–64	70	13	18.6	0.41	0.21–0.82	0.43	0.20–0.89
Chi^2^(1) for trend = 23.3, p < 0.001							
**Smoking status**							
Non–smoker	980	227	23.2	1	–		
Ex–smoker	138	37	26.8	1.09	0.72–1.64		
Current	125	34	27.2	1.15	0.75–1.77		
**Marital status**							
Married	1,027	234	22.8	1	–	1	–
Separated/divorced/widowed	101	28	27.7	1.68	1.04–2.73	1.29	0.71–2.32
Single	116	36	31.0	1.30	0.84–1.99	1.10	0.47–2.57
**Age at 1st sexual intercourse (yrs)**							
<17	107	29	27.1	1	–		
17–19	518	119	23.0	0.80	0.49–1.29		
>19	602	144	23.9	0.84	0.52–1.35		
Chi^2^(1) for trend = 0.18, p = 0.42							
**Number of full–term pregnancies**							
Nulliparous	13	6	46.2	2.17	0.70–6.71		
1	263	73	27.8	1	–		
2	318	72	22.6	0.84	0.57–1.24		
3	220	49	22.3	0.88	0.56–1.37		
≥4	323	59	18.3	0.72	0.46–1.13		
^#^Chi^2^(1) for trend (excluding nulliparous) = 3.80, p = 0.05							
**Use of hormonal contraceptive**							
Never	681	173	25.4	1	–		
Past	352	79	22.4	0.86	0.63–1.17		
Current	210	46	21.9	0.64	0.44–0.95		
**Husband’s extramarital sexual relationships**							
No	849	178	21.0	1	–	1	–
Yes	214	63	29.4	1.69	1.20–2.38	1.66	1.17–2.34

*Adjusted for age and the other variables in the table. Women were excluded from the models (e.g. smoking status) if they were missing the variable, so the different models included different numbers of women. ^#^Trend estimated excluding nulliparous women.

## Discussion

This is the first HPV prevalence survey to be conducted in Fiji, a country with high cervical cancer rates. It revealed an important prevalence of HPV (24.0%) in the general female population, similar to that found in the only other HPV prevalence survey conducted (to date) in the Pacific, in Vanuatu [9] (although surveys have also been conducted in specific populations in some countries: Hawaiian women [10] and indigenous women in Australia [11]). The survey in Vanuatu [9] found a similar age-standardised prevalence of 25.0%. Findings in Fiji can be validly compared to those in Vanuatu and in the other ~25 IARC HPV surveys, for which only Guinea (51.5%) [12] and Mongolia (35%) [13] showed higher prevalence, since they are based on the same study design and HPV testing methods.

HR HPV prevalence across the IARC HPV prevalence surveys has been shown to be strongly correlated with cervical cancer incidence rates [14]. This correlation is particularly strong for HR HPV prevalence among women aged 55-64 [14], and a HR HPV prevalence of 10% in this age group in Fiji would be predictive of the observation of a high cervical cancer risk (51 cases per 100,000 person years) [4].

HPV16 was the most frequently detected HPV type. This is consistent with a meta-analysis showing that HPV16 is the most common cervical infection worldwide in all grades of cervical diagnoses [15]. The distribution of the four most common cervical HR HPV genotypes in our study was HPV16, 52, 56 and 59 identical to those reported in Vanuatu [9]. HPV18 was not found as commonly in the current study as in other studies in Asia or North America [15], a phenomena that was also observed in Vanuatu [9].

The only risk factor found to be significantly positively associated with cervical HPV infection in the multiple-adjusted analysis was the husband’s extramarital sexual relationships (OR = 1.66; 95% CI, 1.17-2.34). However, we did not have detailed information on the women’s sexual behaviour as it was considered inappropriate to ask questions about number of sexual partners to Fijian women in the current study. A positive association with being separated/divorced/widowed was present in the age-adjusted analysis and it was consistent with findings in previous IARC surveys [16]_._ Notably, significantly higher HPV prevalence was found in single/separated/divorced women aged 45 years or older than in their married counterparts. This difference is likely due to relatively high-risk sexual behaviour in this group of unmarried middle-aged women or in their male sexual partners, i.e., a feature that may vary according to the age assortativity of sexual partnerships in different populations [17].

The strengths of this study include the relatively large number of women surveyed and the comparability of study methods and our findings with those of other IARC surveys [12,18-21]. The main limitation of the study was that we were unable to obtain a genuinely random population sample. This occurred because of the absence of a reliable residential list, and a lack of specific postal addresses, as well as the mobility of the population. This led to the inclusion of women who worked in the study area, but who did not necessarily live there. However, it is unlikely that the non-representativeness of the sample had a major impact on the study findings since the HPV prevalence was similar whether or not we included the women that lived outside of the study area (data not shown). Although the exact participation rate for the study is difficult to estimate, the age distribution of the participating women was similar to the age distribution of Fiji women in the latest census (2007), except for an under-representation of women aged 16 to 24 years (12% in our study compared with 29% among Fiji’s female population [5]). Another limitation was that it was impossible to acquire detailed information on a woman’s sexual behaviour.

Our study found that only 1% of Pap smears were abnormal, which is much lower than would be expected given the high HPV prevalence and from previous reports in Fiji where one in 10 Pap smears were abnormal [6], and in Vanuatu where 13.6% were abnormal [9]. The majority of the cervical abnormalities in our study were classified as high-grade lesions but the classification could not distinguish benign cellular changes from atypical squamous cells of undetermined significance (ASCUS). This contrasts with previous studies in Fiji and Vanuatu that found more low-grade abnormalities [6,9]. These findings highlight, once again, inherent problems in achieving reproducible standards of cytological screening in settings where large quality-assured cervical cancer screening programmes are not in place [22]. Furthermore, a large number of women with an abnormal cytology result did not return for follow-up (11 out of 13). This phenomenon was also reported in recent studies in Vanuatu [9] and Fiji [6]. The Fijian population undertakes a large amount of movement between family groups and residencies, giving rise to a dynamic and mobile population. This is a significant cause of the failure to reach clients to inform them of their results and the need to return in a timely manner for further management/treatment. The sharing of a few common names among clients is also a real issue since it is difficult to accurately identify individuals. It is routine and necessary practice in Fiji laboratory request forms to record a client's father’s name (in Indian Fijian clients) in order to help identify individuals. It has been recommended [8] that all clients to be assigned a unique identifier, to assist in identifying patients and tracking test results to assist with follow-up. Due to high workloads, nurses often view searching for patients to inform them of their smear results and to arrange follow-up as a poor use of their time [6].

## Conclusion

Cervical cancer is the most common cancer for women in Fiji. Our findings provide essential information for future policy decisions on the most appropriate methods of cervical cancer prevention in Fiji and in the Pacific region as a whole, particularly in light of the recent development of vaccines against HPV16/18 (which account for 77% of cervical cancer in Fiji [8]) as well as cheap and simple HPV-based screening tests designed for low- and middle-income settings [23]. A relatively high prevalence of HR HPV (10%) in women older than 45 suggests a strong incentive not to exclude older women from at least the first screening round in high-risk and previously unscreened populations. The choice of HPV-based primary screening is especially valuable in these older women in some low-resource countries on account of the loss of sensitivity of visual inspection techniques as the squamous–columnar junction withdraws into the endocervix [24,25]. Difficulties in following-up screen-positive women in such a high-risk population, also point to the strong need for access to a rapid HPV testing method that allows see-and-treat approaches at the same visit. An on-going active education campaign on the health burden due to cervical cancer (a condition that is very much preventable through early detection of pre-cancerous lesions) and the rationale for regular cervical screening and follow-up for the general population and health workers would also be highly beneficial.

## Materials and methods

At the beginning of every interview and prior to specimen collection, individuals were again given one explanation of the objectives and aims of the study where they gave consent. The study was approved by the National Health Research Committee of the Ministry of Health of Fiji and was carried out between July 2010 and February 2011.

### Participants

No reliable resident list was available for the study area. We concentrated, therefore, on invitations through community meetings, church leaders, public health outreach and pamphlets at village shops. We conducted Pap smears and obtained specimens for HPV testing in neighbouring villages as well as in the Valelevu Health Centre. In addition, workers at a garment factory, situated in the study area (at least half of the employed women are local), were included in the study.

A standardised questionnaire, similar to that used in other IARC-coordinated HPV surveys [9], was administered to all study participants by trained interviewers. The questionnaire included questions on socio-demographic characteristics, sexual behaviour of the women and of their partners, reproductive factors, use of contraceptive methods, and smoking habits.

### Sample collection

During community meetings, and individually prior to sample collection, clients were given an information sheet about the study, which was also explained in English, Fijian or Hindi by one of the public health nurses. The nurses also did the Pap smears and took the specimens for HPV testing.

The cervical cell sample collection methodology has been described elsewhere [13]. Briefly, after the preparation of a Pap smear, the brush containing cellular material was placed in a vial containing PreservCyt media (Hologic, Bedford, MA, USA). The brush was fully rinsed and shaken in the media to remove any residual cells and the brush discarded. Cell samples in the PreservCyt media were transported to the Fiji Ministry of Health Viral Laboratory the same day and stored at 4°C until air freighted to IARC (Lyon, France) within six weeks of collection of the specimen.

### Cytology

The Pap smears were read and classified at the main tertiary hospital (Colonial War Memorial Hospital, Suva) laboratory of Fiji. During a one month period between September and October 2010 (due to acute laboratory staff shortage) 70 smears were sent for reporting at Medlab Pathology, Auburn, Australia which collaborates with the Fiji laboratory. Women with visible abnormalities or abnormal cytology were managed according to local protocols.

### HPV testing and genotyping

HPV DNA analysis was performed at the VU University Medical Centre, Amsterdam, The Netherlands according to a protocol similar to that for previous IARC HPV prevalence surveys [9,18]. Beta-globin polymerase chain reaction (PCR) analysis was performed first to confirm the presence of human DNA in all specimens [19]. Cervical specimens were subject to HPV DNA genotyping, as described elsewhere [20]. HPV positivity was assessed by hybridisation of PCR products in an enzyme immunoassay (EIA) using two HPV oligoprobe cocktails that, together, detect the HPV types outlined in Table 1. In addition, HPV positivity was assessed by Southern blot analysis of PCR products with a cocktail probe consisting of specific DNA fragments under low stringency conditions, allowing the detection of HPV types not included in the EIA probe cocktails. Subsequently, GP5+/6+ mediated PCR was repeated on positive samples in triplicate to generate sufficient products for typing. After pooling of these PCR products, typing was performed using EIA and HPV type-specific oligoprobes for the high-risk (HR) and low-risk (LR) types described above. HPV types considered HR for this analysis were 16, 18, 31, 33, 35, 39, 45, 51, 52, 56, 58, 59, and 68 [9]. All other types were considered LR.

### Statistical analysis

Age-standardised HPV prevalence was estimated to allow comparisons with other IARC surveys [12], using five-year age-groups and the World standard population [26]. Odds ratios (ORs) for HPV positivity (and 95% confidence intervals (95% CIs)) were estimated using logistic regression. ORs were adjusted for age in the initial analyses, and then also for exposure variables which showed elevated risks in preliminary analyses.

## Abbreviations

ASC-H: Atypical squamous cells – cannot exclude high-grade squamous intraepithelial lesion; ASCUS: Atypical cells of undetermined significance; EIA: Enzyme immunoassay; IARC: International agency for research on cancer; OR: Odds ratio; HPV: Human papillomavirus; HR: High-risk; LR: Low-risk; PCR: Polymerase chain reaction.

## Competing interests

The authors declare that they have no competing interests.

## Authors’ contributions

SF led the daily field work, data collection and data entry, and contributed to the literature review and writing of manuscript. NB undertook the data analysis, produced the tables and figures, and contributed to the writing of the manuscript. NP provided a major contribution to the study design, data analysis and writing of the manuscript. PJFS & CJLMM undertook the HPV typing and DNA analysis of the cervical cell specimens. LW contributed to the survey field work and specimen collection at the health clinics. GC contributed to the development of the proposal, the literature review, data analysis and writing of the manuscript. SF contributed to the development of the proposal, the literature review and writing of the manuscript. All authors read and approved the final manuscript.
